# Net financial benefits of averting HIV infections among people who inject drugs in Urumqi, Xinjiang, Peoples Republic of China (2005–2010)

**DOI:** 10.1186/1471-2458-12-572

**Published:** 2012-07-29

**Authors:** Mingjian J Ni, Li Ping Fu, Xue Ling Chen, Xiao Yuan Hu, Kim Wheeler

**Affiliations:** 1 Xinjiang Uighur Autonomous Regional Centre for Disease Control HIV/AIDS and Sexually Transmitted Infection Prevention and Control Center, 48 Beijing South Road, Urumqi, Xinjiang Uighur Autonomous Region, Peoples’ Republic of China

**Keywords:** HIV infections averted, Net financial benefit

## Abstract

**Background:**

To quantify the contribution of locally implemented prevention programmes in contributing to reductions in treatment and care costs by averting HIV infections among those who inject drugs this study calculates net financial benefit of providing harm reduction programmes using information from services being implemented in Urumqi, Xinjiang Uighur Autonomous Region of China ( between 2005 and 2010).

**Methods:**

Information was collected to assess cost of providing methadone treatment (MMT) and needle and syringe programmes (NSP). HIV incidence was estimated among people who inject drugs (PWID). HIV infections averted were calculated. Net benefit was assessed by estimating costs of providing prevention programmes and comparing these to the costs of providing care.

**Results:**

An estimated 5678 (range 3982–7599) HIV infections were averted between 2005 and 2010 and the net financial benefit of providing harm reduction programmes compared to treatment and care costs for HIV infections averted was USD 4.383 million during the same time period.

**Conclusion:**

These results demonstrate the net and accumulating benefit of investing in harm reduction programmes for PWID in Urumqi. The return on investment progressively increased during the time period studied and it is clear that these cost savings will continue to accrue with the continued implementation of HIV prevention interventions in the community that include harm reduction programmes targeted at PWID.

## Background

Over the last several years increasing numbers of studies have sought to demonstrate positive benefits of preventing transmission of blood borne infections including HIV through implementation of harm reduction programmes for people who inject drugs (PWID). In 2001, a country wide analysis was carried out in Australia to project a return on investment of implementing needle and syringe programmes (NSP) in preventing transmission of HIV and Hepatitis C [1]. Subsequent small and large scale studies undertaken in a variety of settings have all shown positive health outcomes and significant cost savings as has a 2009 follow-up study in Australia [2]. Methadone maintenance therapy (MMT) programmes have also been evaluated for their impact on preventing HIV transmission and reducing crime rates [3,4]. However there are very few studies arising from China examining the financial benefits of combined harm reduction programmes (NSP and MMT) for PWID.

HIV among PWID was first identified in China in 1989. Since that time more than 370,000 people have been identified as living with HIV. Injecting drug use remains a common transmission route particularly in Yunnan province and Guangxi Zhang and Xinjiang Uighur Autonomous Regions.

The Xinjiang Uighur Autonomous Region (XUAR), in the northwest of the People’s Republic of China, ranks fifth in HIV prevalence in China. Between 1996 and the end of December 2010 33,149 people in the region had been identified as HIV positive (9% of the total number of people with HIV in China) (People’s Republic of China National HIV and AIDS Case Reporting 2010). Of these PLHIV, 54.7% reported having injected drugs.

In XUAR, high rates of sharing injecting equipment have been found in the absence of harm reduction programmes. In 2001, research in 20 counties and cities in XUAR showed that 69.5% of drug users had injected drugs and 61.2% of them reported sharing injecting equipment. The rate of sharing in some specific regions such as Yining City was as high as 83.9%. Operational research in 2006 to evaluate the impact of harm reduction programmes funded by the China-Australia Xinjiang HIV/AIDS Prevention and Care Project (XJHAPAC) showed that between 50-70% of people who injected drugs reported having shared needles and syringes. At the end of 2008 those rates had dropped to 20-40%.

Harm reduction programmes began in XUAR in 2004 with a pilot NSP in Yining City (an area in the north of XUAR significantly affected by HIV amongst PWID). The programme was expanded in 2006 to Urumqi (capital city of XUAR) and Kashgar City (southern XUAR) and subsequently to many areas of XUAR known to have high numbers of PWID. These interventions were initially financed by international donor funds and, from 2009 onwards, by national and local government funding.

MMT began in XUAR in Yining, Urumqi and Kashgar Cities in 2005 and since that time have been expanded into many areas of XUAR. The national government funded the establishment of the initial clinics and MMT continues to be funded through national and local government contributions.

This paper explores the net financial benefit in Urumqi of providing harm reduction programmes in averting HIV infection as a consequence of injecting drug use as compared to the costs of providing HIV treatment and care. Urumqi was chosen as the site for this research based on completeness of records and readiness of access to staff. This study draws on available information about the incidence of HIV among PWID between 2000 and 2010 and specific studies to assess costs.

## Methods

Numbers of community based service users were collected from existing reporting systems for NSP sites and MMT clinics in Shuimogou, Shayibake and Tianshan Districts of Urumqi. The costs of implementing harm reduction programmes were collected from financial records and a survey of staff from these same sites. The survey collected information for 2008–2010 for MMT and 2006–2009 for NSP.

Costs were assessed based on establishment costs (equipment, staff training), operational fixed costs (rent, utilities, equipment maintenance, staff salaries) and operational variable costs (in-service staff training and consumables for testing; supplies such as methadone, needles and syringes and condoms). These cost definitions are generally accepted as describing direct costs of services [5]. Income from national and local government, donor and service user payments covered the costs of providing MMT services.

Costs for MMT were estimated for 2006 and 2007 based on cost per service user derived from the 2008 costs. Costs for NSP in 2010 were estimated based on costs per service user in 2009.

Trends in incidence were measured by BED HIV-1 EIA Assay testing. Blood samples were tested from drug users resident at the Urumqi City Public Security Bureau Detoxification Centre from 2000 to 2010. From 2000–2005, BED HIV-1 EIA Assay testing was carried out on stored blood samples and from 2006–2010 on fresh samples. Obtaining blood samples from all residents in Detoxification Centers is a routine component of admission and required for the regional surveillance system. Residents were informed of the reasons for blood sampling. Informed consent for the use of stored blood samples was not possible to obtain due to the lack of accurate and current contact information for those who had given samples between 2000 and 2005.

To estimate PWID community based population size, study results from previous internal studies were used. In 2010, an internal XUAR Centre for Disease Control research study showed that 68.2% of 2487 drug users in various communities including Urumqi, reported having injected opiates. Therefore, the population size of PWID was estimated based on numbers of registered (10,481) and estimated total drug users (20,000) in Urumqi provided by the XUAR Public Security Bureau. The minimum number of registered PWID was 7,148 (68.2% of 10,481) and the maximum number was 13,640 (68.2% of 20,000). These estimates were used to establish the upper and lower ranges for HIV infections averted in each year of the study.

To estimate HIV infections averted incidence was projected for 2006 to 2010 based on the average rate of increase as measured in samples between 2000 and 2005 using a standard average rate of increase formula [6]

(1) $$\overset{―}{x}=n\sqrt{\frac{a_{1}}{a_{0}}x\frac{a_{2}}{a_{1}}\times ....\times \frac{a_{n}}{a_{n-1}}}=n\sqrt{\frac{a_{n}}{a_{0}}}$$

$\bar{x}$–average rate of increase

n–number of years in the series

a_0_–origin of series (year)

a_1_–first figure in series (year).

Annual projected incidence results were compared to actual results on tested blood between 2005 and 2010 and the difference between these results enabled estimations of infections averted.

Costs of providing treatment and care were calculated based on personal costs for PLHIV and government supported costs. Personal costs for PLHIV were estimated from survey results of 100 PLHIV in Urumqi (36 participants were using antiretroviral therapy (ART) and 64 were not). Government costs were summated based on testing costs, provision of ART, treatment costs of opportunistic infection and follow-up service delivery. The government supported costs for providing free treatment and other services were added to the personal costs to derive the total direct costs of treatment.

Calculation of annual and cumulative net benefit was based on annual costs of providing harm reduction programmes which were derived from the average costs of treatment services. This provided the basis for the derivation of estimated net financial benefit and a ratio of comparative investment (USD1 spent on harm reduction saves USDX spent on treatment).

All data were input to EpiData (Version 3.1) and analysis was completed with SPSS (Version 11.0). All information used in this paper was provided by the Xinjiang Uighur Autonomous Regional Center for Disease Control. Information ascertained through surveys was provided freely and openly by survey participants.

The XUAR Bureau of Health Ethics Committee was established to support the implementation of the China National Bureau of Health Research Programme. The information collection and methodologies used to support this study were approved by this group.

## Results

### Service users

The number of users of harm reduction services is given in Table 1. These figures capture the number of people who used NSP services at least four times during a year or continued to use MMT services throughout a year. For the purpose of this study it has been assumed that all service users have an opportunity to learn basic information about HIV and repeated service contact indicates intention to change risk behaviors.

**Table 1:** Numbers of people who inject drugs using harm reduction services

	**2006**	**2007**	**2008**	**2009**	**2010**
Methadone maintenance therapy (MMT)	1407	2430	2569	2111	1852
Needle and syringe programmes (NSP)	913	1303	1298	1669	1556
**Total**	**2320**	**3733**	**3867**	**3780**	**3408**

The numbers of users of services increased annually over the time period studied from 2320 in 2006 to 3867 in 2008, declining to 3408 in 2010. A variety of reasons, including funding changes and social issues, account for the 11.8% decline in service user numbers between 2008 and 2010 . Reductions in funding for the NSP changed the arrangement of outreach service provision and at many sites outreach worker numbers were reduced and linked to MMT. Social unrest in Urumqi in 2009 and subsequent increased police activity also reduced the numbers of contactable PWID in the community.

### Costs of harm reduction programmes

Costs of NSP were collected from sites for 2006–2009 and 2010 costs were estimated based on costs per service user in 2009. Significant variation in financial input to NSP is due to large injections of funding from XJHAPAC to establish NSP sites in 2006 and to expand services and coverage in 2008. The Global Fund also supported activities over that period (2006–2008). It is therefore assumed that the financial inputs in 2009, when both of these external funding sources stopped, are more closely aligned with actual current costs of operation of NSP sites. Therefore, the 2010 costs per service user were assessed based on 2009 costs per service user of 470.28 RMB (USD72) (Table 2).

**Table 2:** Estimated costs of harm reduction services in Urumqi

	**2006**	**2007**	**2008**	**2009**	**2010**
Total cost of MMT (RMB)	1,715,133*	2,962,170*	3,131,538	3,429,569	3,217,224
Total cost of NSP (RMB)	1,697,101	1,027,948	2,152,454	784,905	731,756*
Total (RMB)	3,412,234	3,990,118	5,283,992	4,214,474	3,948,980
Total (USD)**	**$524,959**	**$613,864**	**$812,922**	**$648,381**	**$607,535**

* Estimated costs **USD1 = 6.5RMB.

Costs of providing MMT were collected by survey at participating sites for 2008–2010. Costs for 2006 and 2007 were estimated based on costs per service user in 2008 (Table 2). Average cost per year was USD641,532 with costs being highest in 2008 when investment in both services was USD812,922 to facilitate expansion of services.

### Estimating HIV infections averted

#### HIV incidence among PWID

**Figure 1:**
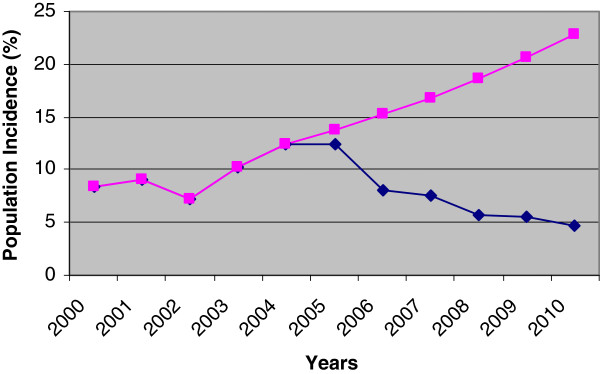
Incidence of HIV among PWID.

BED HIV-1 EIA Assay testing was carried out on samples collected as part of the national sentinel surveillance. Between 2000 and 2010 14,524 blood samples from people who inject drugs were tested. Of these 4740 were found HIV positive, 2628 were included in BED HIV-1 EIA Assay (after exclusion of duplicate samples and those already in the case reporting system) and 504 new cases of infection were confirmed. Annual HIV incidence (based on the China correction coefficient) was determined (Figure 1). From 2000 to 2005, (pre-service period) 62.2% of the blood samples in the series were analysed and from 2006 onwards the remaining 37.8% were included in the analysis.

The incidence trends indicated that, between 2000 and 2004, incidence increased gradually (8.37% to 12.49%) and from 2005 onwards, incidence declined from 12.42%, to 8.03% in 2006 and 4.63% in 2010 (Figure 1). The most significant decline in incidence appears to be from 2005 to 2008 which also correlates with an increase in users of the community harm reduction programme (Table 1).

It should be noted that sample size, after correction, in 2008 (627), 2009 (1022) and 2010 (303) was significantly lower than other years (where sample size ranged between 1100 and 1800 per year) due to pressures of work for the Center for Disease Control (CDC). However, it appears that the whole series tends to show a decline in incidence from 2005 (Figure 1).

Harm reduction programmes were introduced in Urumqi in 2005 and augmented from 2006 onwards by continuous community-based HIV prevention promotion activities and outreach services. On this basis it is concluded that the programmes included in this study, being the only services of this type in Urumqi, have, in part, contributed to reducing incidence of HIV among PWID.

#### HIV infections averted

Using the difference between projected incidence and BED HIV-1 EIA estimated incidence it was then possible to derive the number of HIV infections averted (Table 3). Average HIV infections averted between 2005 and 2010 are estimated to be 5790 (range 3982–7599).

**Table 3:** Estimated numbers of HIV infections averted among people who inject drugs

	**2005**	**2006**	**2007**	**2008**	**2009**	**2010**	**Total**
Minimum infections averted	78	428	551	814	934	1177	**3982**
Maximum infections averted	148	817	1051	1554	1782	2246	**7599**
**Average infections averted**	**113**	**623**	**801**	**1184**	**1358**	**1712**	**5791**

In calculating these figures the number of PWID is assumed to be static. In addition, the progression of HIV in the drug using group in the absence of prevention services is assumed to increase at the same rate as the population being tested. However, identification of HIV in the population is dependent on testing capacity and accurate case reporting which may mean that the progression of disease within the entire injecting drug using population cannot be clearly defined or projected at any specific time.

### Costs of providing treatment and care

The personal financial costs of being HIV positive were assessed by survey and these were added to the direct costs supported by government for those diagnosed but not on antiretroviral therapy (ART) and those people living with HIV (PLHIV) on ART. The average cost was found to be USD386.30 per year for those not on treatment and USD1147.20 for those on ART.

The results of survival information from within China and internationally were used to estimate annual average costs of treatment and care for the two treatment scenarios. Survival with untreated HIV is 9–11 years and diagnosis usually occurs within 2–5 years [7]. According to information from surveys within China, 76% of PLHIV survive at least 5 years on ART [8]. In some developed countries survival can be extended up to 50 years (average 29 years) [9].

In China, free ART was first introduced in 2004 for those with CD4 ≤ 200. From 2010 onwards the national free treatment scheme was provided for people with CD4 ≤ 350. From 2010, in some selected regions, including XUAR, free treatment was provided from diagnosis for those with CD4 between 350 and 500 (on average two years after infection). For those diagnosed as HIV positive with an HIV negative spouse or regular sexual partner treatment is initiated on diagnosis, irrespective of CD4 level, to assist in preventing transmission. Therefore, costs of treatment (personal and national) could be estimated in two ways:

1. Treatment Cost Scenario 1: Diagnosis at 2 years following infection (no direct costs incurred), 3 years from diagnosis without ART (3 x USD386.30/year) and ART for 5 years (5 x USD1147.20/year). A total of 10 year survival from infection: total cost USD6,895.00 (USD861.90/person/year). This was used for our study.

2. Treatment Cost Scenario 2: ART introduced on or around diagnosis (after two years following infection) with an average of 29 years survival: total cost USD33,269.70 (29 x USD1147.20/person/year) which will be the predominant treatment cost scenario for the future.

### Calculation of annual and cumulative net benefit

The annual and cumulative net benefit of averting HIV infections was calculated using the average HIV infections averted annually. This finding was then used to estimate the cost of treatment for this group based on the annual costs of providing one person with treatment in the two treatment scenarios (Table 4).

**Table 4:** Net benefit of providing harm reduction services from 2006-2010

**A**	**B**	**C**	**D**	**E**
**Year**	**Total costs of HR (USD)**	**Infections averted - cumulative**	**Treatment scenario 1 (USD861.90/person/year)**	**Net benefit (10 year survival) cumulative (USD)**
2006	524,959	736	634,358	109,399
2007	613,864	1537	1,324,740	710,876
2008	812,922	2721	2,345,230	1,532,308
2009	648,381	4079	3,515,690	2,867,309
2010	607,535	5791	4,991,690	4,383,728
Total	3,207,661			

Table 4 shows that from 2006 to 2010 there were progressive and positive increases in net benefit of averting HIV infections treated under Treatment Scenario 1 from USD109,399 in 2006 to USD4,383,728 in 2010.

In 2010 the new national policy to provide early treatment began implementation (Treatment Scenario 2) but was not introduced to XUAR until the end of 2011. If this policy had been introduced in 2010 in XUAR it can be seen that the treatment costs saved would increase if all HIV infections averted in 2010 (1712) were treated under treatment scenario 2. The accrued net benefit or return on investment for the five year period up to 2010 would be USD 4,872,212 which is an additional cost saving of USD488,484. This was calculated based on the sum of the accrued costs of the two treatment scenarios existing simultaneously in 2010. Therefore, the net benefit is expected to rise significantly as people entering the treatment programme are treated under treatment scenario 2.

**Figure 2:**
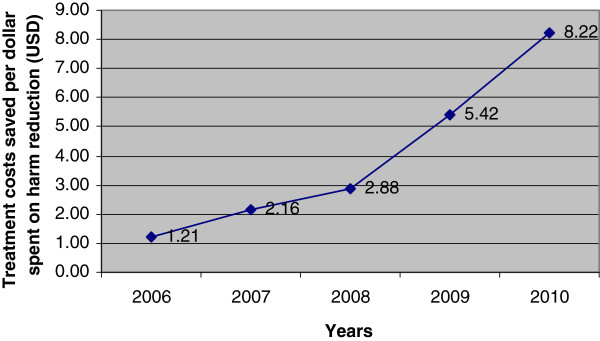
Ratio of investment in harm reduction as compared to treatment costs.

The calculation of costs saved in providing treatment per dollar invested in harm reduction programmes demonstrated progressive cost benefits over the period of study. This is shown in Figure 2 and was calculated annually (Table 4, Column B: Column D) for Treatment Scenario 1. This ratio increased from USD1 (invested in harm reduction): USD1.21 (saved in treatment costs) in 2006 to USD1:USD8.22 in 2010.

In calculating this ratio for the first year (2010) of Treatment Scenario 2 the ratio was 1:3.2. If treatment Scenarios 1 and 2 were to exist together the cost ratio for 2010 would be USD1:USD9. Therefore, the return on investment will increase as survival rates increase (with implementation of Treatment Scenario 2) assuming cost components remain relatively stable, that HIV infections averted continue to increase and incidence remains stable or decreases.

These results demonstrate the net and accumulating benefit of investing in harm reduction programmes for PWID in Urumqi. The return on investment has been progressively increasing during the time period studied and it is clear that these cost savings will continue to accrue with the continued implementation of HIV prevention interventions in the community that include harm reduction programmes targeted at PWID.

## Discussion

This paper reports an assessment of return on investment based on calculation of net benefit in providing drug related harm reduction programmes in Urumqi between 2006 and 2010 based on estimates of HIV infections averted. Over this time period it is calculated that an average of 5790 (range 3982–7509) HIV infections have been averted among PWID. It is possible that the provision of services analysed in this paper specifically targeted at preventing HIV transmission in this group have contributed, in part, to this given that these are the only services available to community based PWID in Urumqi. The costs of implementing harm reduction programmes (MMT and NSP) were calculated and the net amount saved in treatment costs was estimated to be USD 4,383,728,212 accrued over the period studied. The ratio of return on investment was calculated and this increased annually from USD 1 (invested in harm reduction): USD1.21 (saved in treatment costs for Treatment Scenario 1) in 2006 to USD 1: USD 8.22 in 2010.

Health economic analysis of the impact of harm reduction programmes in preventing HIV in China is limited. At least one study, in Yunnan, has appeared in the literature analyzing the benefit of NSP. [10] reported that between 2002 to 2008, the numbers of PWID protected from HIV was estimated between 5200–7500 with cost savings of USD 1.38-1.97 million per year. The results of this research in Yunnan tend to support the findings of this research in Urumqi even though the methodologies applied are different.

The net benefits may be underestimated. Only direct costs of providing harm reduction programmes and care and treatment services have been taken into account. Indirect benefits such as the ability of methadone users to return to work and the social benefit of reported reductions in crime; and, long-term treatment for PLHIV contributing to their capacity to participate fully in society and generate income was not factored into this analysis.

Information on the actual situation is challenging to obtain and this has limited the scope of this study. Information was collated from various sources including surveillance data, specific surveys and on-going studies and research programmes being undertaken in Urumqi. While the quality of data generated through national and local data collection sources is improving, the reliability of information from these sources is still indicative rather than empirical.

Another limitation of the analysis is that BED HIV-1 EIA Assay was used to estimate incidence. BED HIV-1 EIA Assay testing is regarded as overestimating incidence [11,12]. There are few studies in XUAR to verify the incidence rates reported however research in Urumqi in 2003–2005 assessed sero-incidence of HIV among 508 PWID living in the community. This study found that there was a sero-incidence of 8.8 per 100 person years [13]. Therefore, at least for this period, the results of the BED HIV-1 EIA Assay may be, at least, representative of the actual situation.

## Conclusion

Debate is on-going in China over the relative merits of different forms of drug related harm reduction and HIV prevention methodologies [14,15]. In many countries reducing sharing of injecting equipment through the implementation of NSP, drug substitution such as the provision of methadone and buprenorphine and early treatment for PLHIV to reduce risk of transmission co-exist. However, in China due to limited political and social acceptance of drug use and a slow response to HIV in the early stages of the epidemic, the burgeoning number of PLHIV requiring ART has led to current resource allocations and priorities being focused on early treatment. Advocacy for programmes targeted at harm reduction among active injecting drug users is challenging. This is even more so in resource constrained environments and where there is social marginalization of the target population. Against this background , the promotion of research results that indicate the effectiveness of interventions in harm reduction and HIV prevention can support necessary policy shifts and the continued scaling up of successful programmes.

It is recognized that the quality of the data has required significant assumptions to be made in quantifying net benefit of harm reduction programmes targeted at PWID in Urumqi. Nonetheless, the analysis shows an increasing and positive impact in relation to net benefits (return on investment) of all HIV related programmes that avert HIV transmission and, in particular, those programmes targeted at PWID. Continuation and scaling up of these programmes is essential in continuing to prevent HIV transmission.

## Endnotes

^a^Source: Internal report of the Xinjiang Uighur Autonomous Regional Center for Disease Control (2001).

^b^Source: “*Supporting the Development of a Response to HIV”* Final Report for China-Australia Xinjiang HIV/AIDS Prevention and Care Project (2009).

^c^Source: XUAR Center for Disease Control Report for the National Research Programme 2010.

^d^Source: XUAR Public Security Bureau 20019.

^e^USD 3,515,690 (4079 people x USD861.90/year) + USD 1,964,058 (1712 people x USD 1147.20) = USD 5,499,748 less harm reduction costs in 2010 (USD 607,535) = USD 4,872,212.

## Abbreviations

AIDS, Acquired Immunodeficiency Syndrome; ART, Antiretroviral Therapy; CD4, Cluster of Differentiation 4; CDC, Center for Disease Control; HIV, Human Immunodeficiency Virus; MMT, Methadone Maintenance Therapy; NSP, Needle and Syringe (exchange) Programme; PLHIV, People Living with HIV; PWID, People who inject drugs; RMB, Renminbi (Chinese Yuan); USD, United States Dollars; USCDC, United States Center for Disease Control; XJHAPAC, China-Australia HIV/AIDS Prevention and Care Project; XUAR, Xinjiang Uighur Autonomous Region.

## Competing interests

I declare that I and my coauthors have no financial and non-financial competing interests in relation to the submission of this paper for peer review and publication. In the last five years none of the authors have received any reimbursement of fees, funding for this research or salaries from any organization that might gain or loose financially from the publication of this manuscript now or in the future. Funding for the article processing charge is being sourced from research funds provided through the Chinese Ministry of Health. None of the authors hold any stocks or shares in any organization that would gain financially from the publication of this manuscript now or in the future. None of the authors are applying for patents related to the content of this manuscript. There have not been any reimbursements, fees, funding or salary paid to any of the authors by any organization that holds or has applied for patents relating to the content of this manuscript. The authors have no other financial competing interests in relation to the content or publication of this manuscript. In addition, the authors have no political, personal, religious, ideological, academic, intellectual, commercial or other non-financial competing interests in requesting review and publication of this manuscript.

## Authors’ contribution

MJN was the study manager. LPF assisted in promoting the implementation of the study by facilitating contact with all relevant agencies. XLC undertook the development of surveys, supervised the collection of information, collated and analysed the results and reviewed other sources of information to enable the completion of the research. XYH worked with XLC to support the research. KW assisted in the development and submission of this paper. The study results have been presented and discussed with all the authors and the paper has been approved by them.

## Pre-publication history

The pre-publication history for this paper can be accessed here:

http://www.biomedcentral.com/1471-2458/12/572/prepub
